# A Cross-Sectional Study of Iodine Nutritional Status Among School-Age Children in Chongqing, China

**DOI:** 10.3390/nu17050817

**Published:** 2025-02-27

**Authors:** Peng Pang, Jun Xie, Mengping Yang, Shuang Zhou, Yong Zhang

**Affiliations:** 1 School of Public Health, Chongqing Medical University, Chongqing 400016, China; pangpengcq@163.com; 2 Chongqing Center for Disease Control and Prevention, Chongqing 400707, China; 13508308375@163.com (J.X.); ymp1128@163.com (M.Y.)

**Keywords:** iodine nutrition, children, iodine deficiency disorders, goiter

## Abstract

**Objectives**: The aim of the present study was to evaluate the iodine nutritional statuses of children aged 8 to 10 years in Chongqing Municipality in 2023. **Methods**: In this study, we employed multi-stage stratified cluster sampling of non-boarding children aged 8 to 10 years in all 39 counties (districts). The levels of iodine in household salt and those in random urine samples collected from the subjects were tested. In addition, thyroid gland volume was examined using the ultrasound method in subjects from 13 of the counties (districts). **Results**: Of the total 7751 children aged 8 to 10 years selected for inclusion in this study, the median salt iodine concentration (SIC) was 26.7 mg/kg, with an interquartile range (IQR) of 24.2–29.4 mg/kg. The median urinary iodine concentration (UIC) was 226.4 μg/L, with an IQR of 149.5–309.6 μg/L. The median UIC differed significantly between genders and across various regions (*p* < 0.001). The prevalence of total goiter in the children was 2.24% (58/2591), and significant differences were observed in the prevalence of goiter in different body mass index (BMI) groups (*p* < 0.001). The greatest prevalence of goiter was observed in obese children. **Conclusions**: In 2023, children in Chongqing Municipality aged 8 to 10 years as a whole had adequate iodine nutrition and met the national standard for the eradication of iodine deficiency disorders.

## 1. Introduction

Iodine is an important trace element required for the synthesis of thyroid hormones and plays an important role in human growth and development [1]. Chronic insufficient iodine intake may lead to iodine deficiency diseases (IDDs), including goiter, hypothyroidism, intellectual disability, fertility disorders, decreased child survival rates, and growth and developmental abnormalities [2].

In former times, IDDs were highly prevalent in China, as one of the many countries impacted by this issue. The results of surveys conducted in the 1970s showed that IDDs were prevalent to varying degrees in most parts of the country, with approximately 720 million people at risk of iodine deficiency and, at one point, 35 million people affected by endemic goiter [3]. In many endemic areas, 5–15% of children suffered from mild developmental delay (intelligence quotient of 50–69). In order to control and eliminate IDDs, China initiated comprehensive prevention and control measures in 1993, focusing on universal salt iodization, and established a mandatory universal salt iodization program (USI) in 1995 [4]. Since the implementation of the USI, China has made substantial progress in eliminating IDDs. By 2000, such diseases were eradicated at the national level. Monitoring results from 2010 show that IDDs have been eradicated in twenty-eight provinces nationwide (including Chongqing) and almost eradicated in three provinces, namely, Tibet, Qinghai, and Xinjiang [5].

Chongqing is located in southwestern China and is characterized by a general lack of iodine in the natural environment and low iodine content in drinking water [6]. The municipality was once designated as an iodine deficiency disease area. However, after years of comprehensive prevention and control measures based on universal salt iodization, the overall iodine nutritional status of the population has been significantly improved. Based on survey results, the median urinary iodine concentration (MUIC) of children aged 8 to 10 years old decreased from 303 μg/L in 1997 to 221.9 μg/L in 2017, and the goiter rate dropped from 17.42% to 1.93%. Remarkable achievements have thus been made in Chongqing through the implementation of measures for the prevention and treatment of iodine deficiency disease [7].

Although IDDs have been eradicated in Chongqing Municipality, some children remain at risk of iodine deficiency [8]. Continuous monitoring of iodine nutrition is therefore vital. As part of the study presented herein, we conducted a regular survey to evaluate the iodine nutritional status of children aged 8 to 10 years old in Chongqing Municipality in 2023.

## 2. Materials and Methods

### 2.1. Subjects

The present study is a cross-sectional study that involved the use of the Probability Proportionate to Size Sampling (PPS) method based on population size. Based on the requirements of the China Iodine Deficiency Disease Monitoring Program (2016 Edition), 39 counties (districts) were divided into 4 regions based on economic level and geographic environment. The core urban area includes the districts of Yuzhong, Jiangbei, Nanan, Jiulongpo, Shapingba, Yubei, Beibei, Banan, and Dadukou; the western area includes the districts of Bishan, Jiangjin, Changshou, Nanchuan, Fuling, Yongchuan, Hechuan, Qijiang, Tongliang, Dazu, Rongchang, Tongnan, and Wansheng; and the northeastern area includes the districts of Wanzhou, Kaizhou, Liangping, Fengdu, Dianjiang, Zhongxian, Yunyang, Fengjie, Wushan, Wuxi, and Chengkou.

Each monitoring county (district) was divided into five sampling areas based on east, south, west, north, and center. From each sampling area, one township or street was randomly selected. In each township or street, one primary school was chosen, with 40 non-boarding students aged 8 to 10 years selected from each school, ensuring equal gender distribution and balanced age groups. Based on the above sampling design, the total sample size was 7800. When a student was unable to complete the survey, the principle of proximity was applied to find a replacement.

All the study participants and their parents signed an informed consent form to take part in this investigation. This study was approved by the Ethical Review Committee of the Chongqing Municipal Center for Disease Control and Prevention (CDC) (Approval No. KY-2023-032-1), and all the procedures performed in this study involving human participants complied with the ethical standards of the Declaration of Helsinki.

### 2.2. Data and Sample Collection

A questionnaire was used to obtain basic information, such as name, sex, age, and ethnicity. Height and weight were measured uniformly by health professionals using the same type of height- and weight-measuring equipment, with two consecutive measurements and readings accurate to 0.1 cm or 0.1 kg. Samples of approximately 8–10 mL of random midstream urine were collected in EP tubes between 8:30 a.m. and 12:00 a.m. Next, 50–100 g of edible salt from the child’s home was collected and placed in a dry, clean, sealed bag. Due to financial and time restraints, participants from 13 districts (counties) were selected from 39 districts (counties) to carry out thyroid ultrasound examinations on the children.

### 2.3. Measurements

The iodine content of edible salt was tested using the thiosulfate titration method in the “General Test Method for Iodine Determination in Salt Industry” (GB/T 13025.7-2012) [9], and the arbitration law was adopted for Sichuan salt and other fortified edible salts. (Sodium hypochlorite is used to oxidize iodide ions, producing iodate ions, which are then reduced to iodine using potassium iodide. Starch is used as an indicator, and sodium thiosulfate is employed for titration to determine the iodine content.) In our experiments, a 10 g salt sample was dissolved for each iodine determination.

Urinary iodine testing was performed using the health industry standard “Determination of Iodine in Urine Part 1: Cerium Arsenide Catalyzed Spectrophotometric Method” (WS/T 107.1-2016) [10]. Urinary iodine concentration was measured using a MAPADA spectrophotometer (Mapada Instruments Co., Ltd., Shanghai, China).

Thyroid volume was measured using a Mindray M5t ultrasound diagnostic device (Mindray Bio-Medical Electronics Co., Ltd., Shenzhen, China). Thyroid volume measurement followed the “Diagnostic Criteria for Endemic Goiter” (WS 276-2007) [11]. Ultrasound was used to measure the length (L), width (W), and depth (D) of the left and right thyroid lobes, with all the dimensions recorded in millimeters (mm). The volume of each lobe was calculated using the following formula: V (mL) = 0.479 × L × W × D/1000, with the result expressed in milliliters (mL). Total thyroid volume was the sum of the volumes of the left and right lobes, excluding the isthmus.

### 2.4. Determination Criteria

#### 2.4.1. Body Mass Index

The formula for calculating BMI was as follows: BMI = weight (kg)/[height (m)]^2^. Based on the standard of “Comprehensive Evaluation of the Developmental Levels of Children and Adolescents” [12], when the BMI of the examined subject is lower than the BMI of the corresponding age and gender group, they are judged to be emaciated. When the BMI of the subject is greater than or equal to the overweight value of the corresponding age and gender group, and less than the obesity value of the corresponding group, they are judged to be overweight. When the subject’s BMI is greater than or equal to the obese value of the corresponding age and gender group, they are judged to be obese.

#### 2.4.2. Iodized Salt

Based on the determination standard of the SIC of edible salt in Chongqing, 21~39 mg/kg represents qualified iodized salt, <5 mg/kg represents non-iodized salt, and 5~21 mg/kg or >39 mg/kg represents unqualified iodized salt.

#### 2.4.3. Urinary Iodine

Based on the MUIC standard determination recommended in the “Standard Guidelines for the Monitoring of Salt Iodization Program and Evaluation of Iodine Nutritional Status of the Population” proposed by the United Nations Children’s Fund (UNICEF) in 2018, populations with an MUIC < 100 μg/L were defined as iodine-inadequate, those with an MUIC of 100~299 μg/L were defined as having an adequate iodine level, and those with an MUIC of ≥300 ug/L were defined as being in a state of iodine excess. In iodine deficiency, an MUIC of less than 20 μg/L is defined as severe iodine deficiency, whereas MUIC levels between 20 and 49 μg/L and between 50 and 99 μg/L are classified as moderate and mild iodine deficiency, respectively.

In this study, to analyze the subjects’ UIC in detail, we classified their iodine nutritional status according to the MUIC-based evaluation standards established by the World Health Organization, the United Nations Children’s Fund, and the International Council for the Control of Iodine Deficiency Diseases (ICCIDD) in 2007. Populations with an MUIC between 100 and 199 μg/L were classified as having adequate iodine levels, whereas those with an MUIC between 200 and 299 μg/L were categorized as having iodine intake above the recommended level [13].

#### 2.4.4. Thyroid Gland

The determination of thyroid volume in the children was based on the “Diagnostic Criteria for Endemic Goiter” (WS 276-2007). For 8-year-old children, a thyroid volume greater than 4.5 mL was considered indicative of goiter. For 9- and 10-year-olds, the thresholds were 5.0 mL and 6.0 mL, respectively. The epidemiological standards for assessing the severity of IDDs based on thyroid goiter prevalence in school-age children are as follows: a thyroid goiter prevalence of less than 5% indicates sufficient iodine levels; 5–19% represents mild iodine deficiency; 20–29% indicates moderate iodine deficiency; and more than 30% indicates severe iodine deficiency.

### 2.5. Statistical Analysis

Data were statistically analyzed using SPSS 26.0. Normality was tested using the Kolmogorov–Smirnov (KS) test. Measures that did not conform to a normal distribution are expressed as the median (M) and IQR. For continuous variables, the Mann–Whitney U test was used for comparisons between two groups, and the Kruskal–Wallis H test was used for comparisons between multiple groups. The Chi-squared test was used to analyze categorical data, and binary logistic regression was used to estimate the association of goiter incidence with salt iodine and urinary iodine.

## 3. Results

### 3.1. Basic Characteristics of the Subjects

In 2023, 8003 children aged 8 to 10 years were surveyed, 252 children with incomplete information and some missing test results were excluded from the data, and 7751 children aged 8 to 10 years were included in the final analysis, including 3878 boys and 3873 girls. Statistically significant differences were found between the composition ratios of ethnicity, region, and BMI between the thyroid ultrasound examination group and the group that did not undergo this procedure (*p* < 0.001) (Table 1).

### 3.2. Iodine Content in Salt from the Household

The median salt iodine content was 26.7 mg/kg, and the quartiles ranged from 24.2 to 29.4 mg/kg. The coverage rate of iodized salt was 99.00% (7673/7751); in comparison, the consumption rate of adequately iodized salt was 94.75% (7344/7751). The SIC for the minority group was 28.1 (25.7–31.1) mg/kg, while for the Han ethnic group, it was 26.6 (24.1–29.2) mg/kg. Although statistically significant (*p* < 0.001), this difference is considered negligible from an epidemiological perspective. The difference in SIC between region and BMI groups was statistically significant (*p* < 0.001). The differences in the consumption rate of qualified iodized salt among children of different ethnicities (χ^2^ = 8.563, *p* < 0.05) and from different regions (χ^2^ = 10.743, *p* < 0.05) were statistically significant. Details of iodine content in salt consumption by children are shown in Table 2.

### 3.3. Urinary Iodine Concentration

The MUIC of the children was 226.4 μg/L, with quartiles ranging from 149.5 to 309.6 μg/L.

The MUIC of the boys and girls was 235.3 μg/L and 217.0 μg/L, respectively, and the difference between the median UIC of the boys and girls was statistically significant (*Z* = −7.208, *p* < 0.001), with the boys’ median UIC slightly higher than that of the girls.

The MUIC of the Han children was 223.9 μg/L, and that of the ethnic minority children was 257.4 μg/L. The difference in the median UIC among children of different ethnic groups was statistically significant (*p* < 0.001), with ethnic minority children having a higher median UIC than the Han children.

The median UIC of the children varied significantly across different regions (H = 183.026, *p* < 0.001). Pairwise comparisons revealed statistically significant differences in median UIC among all regions except between the core urban area and Southeast Chongqing. No significant differences in median UIC were observed among children of different age groups and BMI categories (*p* > 0.05) (see Table 3 for details).

### 3.4. Thyroid Volume and Goiter Rate

A total of 2591 children’s thyroid volumes were examined, with a median and quartile overall thyroid volume of 3.01 (2.43–3.77) mL, and a total of 58 goiters were detected, with an average enlarged thyroid rate of 2.24%.

There was no statistically significant difference in median thyroid volume among children of different genders or iodized salt groups (*p* > 0.05).

The median thyroid volume differed significantly across age groups (H = 94.086, *p* < 0.001), with thyroid volume increasing as age progressed. The median thyroid volume differed significantly among children within the different BMI categories (H = 107.001, *p* < 0.001). However, pairwise comparisons showed no statistically significant difference in median thyroid volume between the overweight and obese groups (*p* > 0.05). The median thyroid volume showed statistically significant differences across varying UIC levels (H = 15.031, *p* < 0.01), with the largest thyroid volume observed in children whose UIC was ≥300 μg/L.

There was no statistically significant difference in goiter rates in children by age and gender and between iodized salt groups (*p* > 0.05), and the difference in goiter rates (χ^2^ = 26.071, *p* < 0.001) was statistically significant between the different BMI categories, whereby children with obesity exhibited the highest rate of goiter (see Table 4).

### 3.5. Factors Associated with Goiter in Children

Table 5 presents the results of the logistic models regarding the association between goiter prevalence, UIC (both continuous and categorical), and SIC. The findings revealed no significant association between SIC, UIC, and goiter prevalence (*p* > 0.05).

## 4. Discussion

Chongqing is in the hinterland of the Three Gorges reservoir area of the Yangtze River in southwestern China, where iodine deficiency is commonly endemic. Notably, the city was formally an iodine deficiency prevalence area before the implementation of the USI. Zhou Chunpei et al. found that the median iodine content of water in Chongqing was 1.7 μg/L, and the iodine content of drinking water in most areas was low, with water iodine < 10 μg/L accounting for 96.13% of the total, confirming that iodine deficiency is widespread in the natural environment of Chongqing [6]. The USI has been an effective measure to eradicate iodine deficiency caused by natural environmental factors and its subsequent health consequences.

Our study results showed that in 2023, the iodized salt coverage rate for children aged 8 to 10 years in Chongqing was 99.00%, and the consumption rate of qualified iodized salt was 94.75%. Regarding the levels of iodine in the subjects’ urine, in children, UIC < 50 μg/L accounted for 3.46% of the overall samples, UIC < 100 ug/L accounted for 11.28% of subjects, and the prevalence rate of goiter in the children was 2.24%. Based on the data presented above, Chongqing has met Chinese standards for the eradication of iodine deficiency diseases [14].

However, we also found significant differences in median SIC across regions, with the highest median SIC (27.5 mg/kg) observed in Southeast Chongqing, with the consumption rate of qualified iodized salt also being higher than in other regions. Southeast Chongqing is characterized by mountainous terrain, and based on the results of previous studies, groundwater in mountainous areas is not easily enriched with iodine because of its steep gradient, high flow rate, and perennial flooding [15]. Zhou ChunPei et al. also stated in their study that the iodine content of drinking water in Southeast Chongqing is low, at only 1.1 μg/L [6]. The low iodine content of drinking water in Southeast Chongqing is not sufficient to meet the daily iodine needs of residents, and the risk of iodine deficiency is greater than in other regions. Due to these issues, the local government pays considerably more attention to ensuring that qualified iodized salt is available and sold on the market, which may explain the higher median SIC and consumption rate of qualified iodized salt in Southeast Chongqing than in other regions.

In addition, based on the results of a study by Zimmermann et al. [16]—in which the authors found that, in school-age children with an MUIC of 100–299 μg/L, such levels were not associated with thyroid dysfunction—UNICEF has recommended that the range of MUICs for “adequate” iodine nutrition for school-age children be 100–199 ug/L rather than 100–299 μg/L [17]. In our study, the MUIC of 8- to 10-year-old children was 226.4 μg/L, indicating that the overall iodine nutritional status of schoolchildren in Chongqing was “adequate”. However, the MUIC of boys was higher than that of girls, which is consistent with the findings of Wang Zhen et al. [18]. This finding may be due to the differences in intake between boys and girls, with boys’ higher food intake leading to higher daily salt intake [19].

In our study, we also found that thyroid volume varied in different BMI categories, with it being lowest in emaciated children and increasing sequentially in normal-weight, overweight, and obese children. The positive correlation between thyroid volume and body weight was first reported by Hegedüs and colleagues roughly 40 years ago [20]. Similarly, research has shown that thyroid volume is not affected by iodine nutritional status in adults or children but instead increases with body size and anthropometric measurements [21]. However, the factor responsible for the increase in thyroid volume with BMI remains unclear. Some researchers have suggested that obesity may be associated with chronic low-grade inflammation, with ultrasensitive C-reactive protein having been shown to correlate with BMI [22]. Other inflammatory markers, such as tumor necrosis factor, interleukin-1, and interleukin-6, have also been shown to contribute to an increased risk of obesity. All of these cytokines inhibit sodium iodide homotransporter protein mRNA expression and iodide uptake in human thyroid cells, which increases vascular endothelial and tissue fluid exudate permeability and induces local vasodilatation in the thyroid gland, which may lead to abnormal thyroid morphology and function [23]. These events may account for the positive correlation between thyroid volume and BMI. The research demonstrated significant variations in median thyroid volume across different UIC groups (*p* < 0.001), particularly noting that children with a UIC ≥ 300 μg/L exhibited markedly larger thyroid volumes compared to other subgroups. Although UIC may not directly or fully reflect changes in thyroid size and function, it is noteworthy that elevated UIC levels are closely associated with an increased risk of thyroid disorders [24]. Moreover, enlarged thyroid volume may compress the trachea or esophagus, leading to respiratory distress and dysphagia [25]. Therefore, children with increased thyroid volume require closer monitoring of iodine intake, environmental exposures, and lifestyle factors. Prompt medical evaluation with comprehensive assessment and therapeutic intervention is essential to mitigate potential health risks.

Surprisingly, we did not find an association between goiter prevalence and salt iodine and UIC, with two possible explanations for this finding. First, the results of most studies conducted to date show a U-curve relationship between UIC and the prevalence of goiter. In addition to a low UIC, a high UIC can also increase the risk of goiter. Zimmermann et al. showed that thyroid volume begins to increase with a UIC > 500 μg/L [26]. The authors of epidemiologic studies from high iodine-exposed areas in China have found that children with a high UIC have a high prevalence of goiter. For example, in Hebei Province, where children were noted as having an MUIC of 418 μg/L, the prevalence of total goiter in children reached 10.96% [27]. There is widespread iodine deficiency in the drinking water of Chongqing, and the median UIC in children is insufficient to reach 500 μg/L. Second, research has also shown that UIC reflects recent iodine nutritional status, whereas goiter prevalence reflects long-term iodine nutritional status and goiter is not reflected by current iodine intake [28]. Although no association between UIC and goiter prevalence was found in the present study, it is important to determine which type of UIC, low or high, is responsible for the development of goiter in Chongqing.

One limitation of this study is that data on the prevalence of goiter were only collected from children in 13 selected regions of Chongqing. These regions were chosen based on resource and time constraints, which may limit the generalizability of the findings to all children in Chongqing. Additionally, we only collected spot urine samples and did not measure 24 h urine volume, which would have enabled us to calculate 24 h urinary iodine excretion—an indicator independent of hydration status.

## 5. Conclusions

This study demonstrates that children aged 8 to 10 years in Chongqing Municipality exhibited adequate iodine nutrition, as evidenced by a median UIC of 226.4 μg/L, which aligns with China’s national guidelines for iodine deficiency disorder prevention and control. We found that the coverage of iodized salt for children aged 8 to 10 years in Chongqing Municipality reached 99.00%, whereas the consumption rate of qualified iodized salt was 94.75%, underscoring the remarkable success of the USI. Furthermore, sustained and periodic monitoring of the elimination progress regarding IDDs is imperative for preventing both iodine deficiency and excess in children.

## Figures and Tables

**Table 1:** Basic characteristics of the monitoring sample of children aged 8 to 10 years in Chongqing Municipality.

Characteristics	Ultrasound Group *n* (%)	No-Ultrasound Group *n* (%)	Total (*N* = 7751)	*p*
Gender				0.369
Male	1315 (33.9)	2563 (66.1)	3878	
Female	1276 (32.9)	2597 (67.1)	3873	
Age (years)				0.102
8	776 (32.8)	1591 (67.2)	2367	
9	993 (34.9)	1851 (65.1)	2844	
10	822 (32.4)	1718 (67.6)	2540	
Ethnicity				<0.001
Han	2584 (36.7)	4450 (63.3)	7034	
Minority	7 (1.0)	710 (99.0)	717	
Region				<0.001
Core urban area	202 (11.2)	1602 (88.8)	1804	
Western Chongqing	1564 (61.0)	1001 (39.0)	2565	
Southeast Chongqing	825 (37.1)	1401 (62.9)	2226	
Northeast Chongqing	0 (0)	1156 (100)	1156	
BMI (kg/m^2^)				<0.001
Underweight	173 (25.6)	502 (74.4)	675	
Normal	1957 (33.8)	3827 (66.2)	5784	
Overweight	243 (36.4)	424 (63.6)	667	
Obese	218 (34.9)	407 (65.1)	625	

BMI, body mass index.

**Table 2:** Household salt iodine measurement results among children aged 8 to 10 years in Chongqing.

Variables	Total (*N* = 7751) *n* (%)	SIC (mg/kg)	*p*	Iodine-Qualified Salt *n* (%)	*p*
		[M (IQR)]			
Gender			0.473		0.889
Male	3878 (50.0)	26.8 (24.2~29.6)		3673 (94.7)	
Female	3873 (50.0)	26.6 (24.2~29.3)		3671 (94.8)	
Age (years)			0.191		0.414
8	2367 (30.5)	26.8 (24.4~29.5)		2254 (95.2)	
9	2844 (36.7)	26.7 (24.1~29.3)		2685 (94.4)	
10	2540 (32.8)	26.7 (24.2~29.4)		2405 (94.7)	
Ethnicity			<0.001		0.003
Han	7034 (90.7)	26.6 (24.1~29.2)		6648 (94.5)	
Minority	717 (9.3)	28.1 (25.7~31.3)		696 (97.1)	
Region			<0.001		0.013
Core urban area	1804 (23.3)	27.1 (24.8~29.5)		1713 (95.0)	
Western Chongqing	2565 (33.1)	26.0 (23.4~28.6) a		2404 (93.7)	
Northeast Chongqing	2226 (28.7)	26.8 (24.3~29.8) b		2115 (95.0)	
Southeast Chongqing	1156 (14.9)	27.5 (25.0~30.7) abc		1112 (96.2)	
BMI			<0.001		0.147
Underweight	675 (8.7)	26.8 (24.4~29.6)		639 (94.7)	
Normal	5784 (74.6)	26.8 (24.3~29.5)		5496 (95.0)	
Overweight	667 (8.6)	26.3 (24.0~28.8) d		628 (94.1)	
Obese	625 (8.1)	26.6 (23.6~29.2) d		581 (93.0)	

BMI, body mass index; SIC, salt iodine concentration. M, median; IQR, interquartile range. a indicates a statistically significant difference compared to the core urban area; b indicates a statistically significant difference compared to Western Chongqing; c indicates a statistically significant difference compared to Northeast Chongqing; d indicates a statistically significant difference compared to the normal group. All pairwise comparisons were adjusted using Bonferroni correction.

**Table 3:** Urinary iodine measurements in children aged 8 to 10 years in Chongqing.

Variables	Total (*N* = 7751) *n* (%)	UIC (μg/L) [M (IQR)]	*p*
Gender			<0.001
Male	3878 (50.0)	235.3 (160.7~320.2)	
Female	3873 (50.0)	217.0 (139.0~299.2)	
Age (years)			0.977
8	2367 (30.5)	227.4 (149.0~312.9)	
9	2844 (36.7)	226.3 (151.0~308.7)	
10	2540 (32.8)	226.3 (149.0~307.7)	
Ethnicity			<0.001
Han	7034 (90.7)	223.8 (147.5~305.4)	
Minority	717 (9.3)	257.4 (178.9~333.7)	
Region			<0.001
Core urban area	1804 (23.3)	250.0 (171.0~334.2)	
Western Chongqing	2565 (33.1)	221.0 (148.5~318.0) a	
Northeast Chongqing	2226 (28.7)	200.6 (133.8~277.6) ab	
Southeast Chongqing	1156 (14.9)	252.6 (174.8~326.3) bc	
BMI			0.054
Underweight	675 (8.7)	219.7 (142.0~307.4)	
Normal	5784 (74.6)	225.4 (149.1~308.5)	
Overweight	667 (8.6)	237.6 (160.0~316.6)	
Obese	3878 (50.0)	228.4 (152.6~311.3)	

BMI, body mass index; UIC, urinary iodine concentration. M, median; IQR, interquartile range. a indicates a statistically significant difference compared to the core urban area; b indicates a statistically significant difference compared to Western Chongqing; c indicates a statistically significant difference compared to Northeast Chongqing. All pairwise comparisons were adjusted using Bonferroni correction.

**Table 4:** Thyroid volume measurement results and classification of goiter prevalence.

Variables	Total (*N* = 2591) *n* (%)	Thyroid Volume (mL) [M (IQR)]	*p*	Goiter *n* (%)	*p*
Gender			0.978		0.517
Male	1315 (50.8)	3.00 (2.46~3.74)		27 (2.10)	
Female	1276 (49.2)	3.02 (2.39~3.81)		31 (2.43)	
Age (years)			<0.001		0.817
8	776 (29.9)	2.82 (2.30~3.41)		19 (2.45)	
9	993 (38.3)	3.00 (2.38~3.65) a		20 (2.01)	
10	822 (31.7)	3.30 (2.60~4.19) ab		19 (2.31)	
BMI (kg/m^2^)			<0.001		<0.001
Underweight	173 (6.7)	2.61 (2.11~3.12)		2 (1.20)	
Normal	1957 (75.5)	2.97 (2.38~2.69) c		33 (1.70)	
Overweight	243 (9.4)	3.35 (2.61~4.14) cd		5 (2.10)	
Obesity	218 (8.4)	3.41 (2.84~4.43) cd		18 (8.30) cde	
Iodized Salt (mg/kg)			0.549		0.448
Non-iodized salt	17 (0.7)	3.45 (2.41~4.32)		0 (0)	
Adequate iodized salt	2435 (94.0)	3.01 (2.43~3.77)		53 (2.18)	
Inadequate iodized salt	139 (5.3)	2.97 (2.41~3.61)		5 (3.60)	
UIC (μg/L)			<0.010		0.115
<100	303 (11.7)	2.96 (2.32~2.62)		4 (1.32)	
100–200	770 (29.7)	2.94 (2.34~2.73)		12 (1.56)	
200–300	826 (31.9)	3.02 (2.46~3.75)		26 (3.15)	
≥300	692 (26.7)	3.15 (2.58~3.88) fg		16 (2.31)	

BMI, body mass index. M, median; IQR, interquartile range.UIC, urinary iodine concentration. a indicates a statistically significant difference compared to age 8; b indicates a statistically significant difference compared to age 9; c indicates a statistically significant difference compared to the underweight; d indicates a statistically significant difference compared to the normal-weight; e indicates a statistically significant difference compared to the overweight; f indicates a statistically significant difference compared to a UIC > 100 μg/L; g indicates a statistically significant difference compared to a UIC of 100–200 μg/L. All pairwise comparisons were adjusted using Bonferroni correction.

**Table 5:** Association analysis of iodine in salt, urinary iodine, and the prevalence of goiter.

Variables	*N* (%)	Model 1a			Model 2b		
		OR	95% CI	*p*	OR	95% CI	*p*
UIC (ug/L)	58 (2.24%)	1	0.999–1.001	0.858	1	0.999–1.001	0.921
UIC Categories (ug/L)							
<100	4 (1.32%)	Ref.			Ref.		
100–199	12 (1.56%)	1.769	0.587–5.337	0.311	1.725	0.568–5.246	0.336
200–299	26 (3.15%)	1.495	0.702–3.183	0.297	1.459	0.678–3.138	0.334
≥300	16 (2.31%)	0.728	0.387–1.369	0.325	0.730	0.385–1.383	0.334
SIC (mg/kg)	58 (2.24%)	1.011	0.961–1.064	0.669	1.012	0.959–1.068	0.670

Model 1a, unadjusted; Model 2b, adjusted for gender, age, ethnicity, and BMI. Ref. stands for the reference category; OR, odds ratio; CI, confidence interval; SIC, salt iodine concentration; UIC, urinary iodine concentration.

## Data Availability

The original contributions presented in this study are included in this article, and further inquiries can be directed to the corresponding author.
